# The effect of intrinsic subthreshold oscillations on the spontaneous dynamics of a ring network with distance-dependent delays

**DOI:** 10.1186/1471-2202-12-S1-P236

**Published:** 2011-07-18

**Authors:** Fabiano Baroni, Thomas Nowotny

**Affiliations:** 1 Department of Electrical and Electronic Engineering, The University of Melbourne, Melbourne, Australia; 2 Centre for Computational Neuroscience and Robotics, School of Informatics, University of Sussex, Brighton, UK

## 

The spontaneous dynamics of neuronal networks have been studied extensively, due to their critical importance in constraining the information representation and processing that these networks can support when provided with meaningful inputs. However, most studies adopted highly simplified descriptions for the individual neurons and for the delays that occur in the communication between them. In this study, we report the dynamics of a network of excitatory and inhibitory neurons, with different intrinsic properties, spatially arranged on a ring network with distance-dependent delays. We used the Integrate and Fire model (IF) or the Generalised Integrate and Fire (GIF) with subthreshold oscillations as a dynamical description for the individual units. As parameters were varied, we observed a wide spectrum of network activities, including spatially confined network bursts, asynchronous activity, and local or global network oscillations. Small spatial inhomogeneities in neuronal density were greatly amplified by the recurrent dynamics of the network, and resulted in spiking inhomogeneities in the (otherwise spatially symmetric) network. Spatial spiking inhomogeneities were strongly correlated between the excitatory and inhibitory population, even if spatial locations were drawn independently from a uniform distribution. Most importantly, intrinsic dynamical properties of individual neurons greatly affected the spiking distribution across cells resulting from a given spatial distribution. In a salient example, changing the intrinsic properties of the inhibitory population from purely passive to resonant resulted in orthogonal spiking distributions for the same realisation of spatial locations. Efficient information representation and processing require a spiking distribution that is relatively flat across neurons. Small spatial inhomogeneities are likely to exist in the brain. We hypothesise that changes in the density of ionic channels might contribute, along with other forms of plasticity, to the homeostatic maintenance of efficient information processing when spatial inhomogeneities are present.

